# Prediction of mechanical characteristics of shearer intelligent cables under bending conditions

**DOI:** 10.1371/journal.pone.0318767

**Published:** 2025-02-04

**Authors:** Lijuan Zhao, Dongyang Wang, Guocong Lin, Shuo Tian, Hongqiang Zhang, Yadong Wang

**Affiliations:** 1 School of Mechanical Engineering, Liaoning Technical University, Fuxin, China; 2 Liaoning Province Large Scale Industrial and Mining Equipment Key Laboratory, Fuxin, China; 3 Shandong Yankuang Group Changlong Cable Manufacturing Co., Ltd, Jining, China; King Fahd University of Petroleum & Minerals, SAUDI ARABIA

## Abstract

The frequent bending of shearer cables during operation often leads to mechanical fatigue, posing risks to equipment safety. Accurately predicting the mechanical properties of these cables under bending conditions is crucial for improving the reliability and service life of shearers. This paper proposes a shearer optical fiber cable mechanical characteristics prediction model based on Temporal Convolutional Network (TCN), Bidirectional Long Short-Term Memory (BiLSTM), and Squeeze-and-Excitation Attention (SEAttention), referred to as the TCN-BiLSTM-SEAttention model. This method leverages TCN’s causal and dilated convolution operations to capture long-term sequential features, BiLSTM’s bidirectional information processing to ensure the completeness of sequence information, and the SEAttention mechanism to assign adaptive weights to features, effectively enhancing the focus on key features. The model’s performance is validated through comparisons with multiple other models, and the contributions of input features to the model’s predictions are quantified using Shapley Additive Explanations (SHAP). By learning the stress variation patterns between the optical fiber, power conductor, and control conductor in the shearer cable, the model enables accurate prediction of the stress in other cable conductors based on optical fiber stress data. Experiments were conducted using a shearer optical fiber cable bending simulation dataset with traction speeds of 6 m/min, 8 m/min, and 10 m/min. The results show that, compared to other predictive models, the proposed model achieves reductions in Mean Squared Error (MSE), Root Mean Squared Error (RMSE), and Mean Absolute Error (MAE) to 0.0002, 0.0159, and 0.0126, respectively, with the coefficient of determination (R^2^) increasing to 0.981. The maximum deviation between predicted and actual values is only 0.86%, demonstrating outstanding prediction accuracy. SHAP feature analysis reveals that the control conductor features have the most substantial influence on predictions, with a SHAP value of 0.095. The research shows that the TCN-BiLSTM-SEAttention model demonstrates outstanding predictive capability under complex operating conditions, providing a novel approach for improving cable management and equipment safety through optical fiber monitoring technology in the intelligent development of coal mines, highlighting the potential of deep learning in complex mechanical predictions.

## 1. Introduction

Shearer cables, as critical components of the underground power supply system in coal mines, are responsible for transmitting both electrical power and signals [1]. During the reciprocating motion of the shearer, the cables are subjected to frequent bending [2], which alters their mechanical properties, exacerbates fatigue effects, and compromises the stability and safety of the equipment [3]. Therefore, accurately predicting the stress variations in the cores of shearer fiber optic cables is crucial for enhancing coal mine safety, improving equipment reliability, and reducing failure rates.

Existing research on the mechanical properties of fiber optic cables primarily focuses on material characteristics and experimental testing. However, in practical applications, experimental tests often fail to accurately reflect the real-time operational states of cables [4]. With the advancement of machine learning and deep learning, data-driven approaches have demonstrated significant potential in time-series prediction. Although physical and statistical models are well-established, they lack sufficient learning capacity, which limits their predictive accuracy [5, 6]. Machine learning, on the other hand, excels at handling nonlinear relationships and complex data variability [7]. Under complex conditions, deep learning architectures formed by combining multiple algorithms further enhance predictive performance [8].

In recent years, many scholars have carried out a lot of research on fiber optic cables. Ma et al. [9] designed and manufactured a fiber optic composite coal mining machine cable, which provides theoretical support for the manufacture of fiber optic cables for coal mining machines through structural and process optimization. Lv et al. [10] proposed a submarine fiber optic cable fault diagnosis method based on wavelet packet and neural network, which improves the accuracy of submarine fiber optic cable fault diagnosis. Lin et al. [11] established a finite element model of three-core submarine fiber optic cable ship anchor and hooking, analyzed its stress and strain under different working conditions, and studied the change rule of its mechanical properties. Bu et al. [12] proposes a machine learning-based panoramic state monitoring system for fiber-optic composite cables, which is conducive to the operation and maintenance of power transmission lines. Chen et al. [13] proposed a distributed sensing technology based on fiber Bragg gratings to monitor the shape of cables through discrete curvature information. Kou et al. [14] proposed a multi-step prediction method for fiber optic composite cables based on the WLS-SVM, which accurately predicts the operational status of submarine fiber optic cables. Wang et al. [15] proposed a defect detection method for ADSS fiber optic cables based on feature adaptive extraction, which improves the accuracy and efficiency of the detection of galvanic corrosion defects in ADSS fiber optic cables through the improvement of the YOLOv8n model. Lu et al. [16] proposed a Bi-LSTM model based on the VMD and attention mechanism for fault detection of fiber optic composite submarine cables to achieve localization and detection of their fault events. Lian et al. [17] proposed a fiber optic cable service life prediction method based on Bi-LSTM combined with the attention mechanism, capable of accurately predicting the service life of fiber optic cables. Yang et al. [18] proposed a submarine fiber optic cable operation state prediction method based on the attention mechanism and CNN-GRU combined network, which can effectively predict the operation states of submarine fiber optic cable. Li et al. [19] proposed a fiber optic composite submarine cable fault diagnosis method based on VMD and SO-optimized SVM, which improves the fault diagnosis rate.

**Fig 1:**
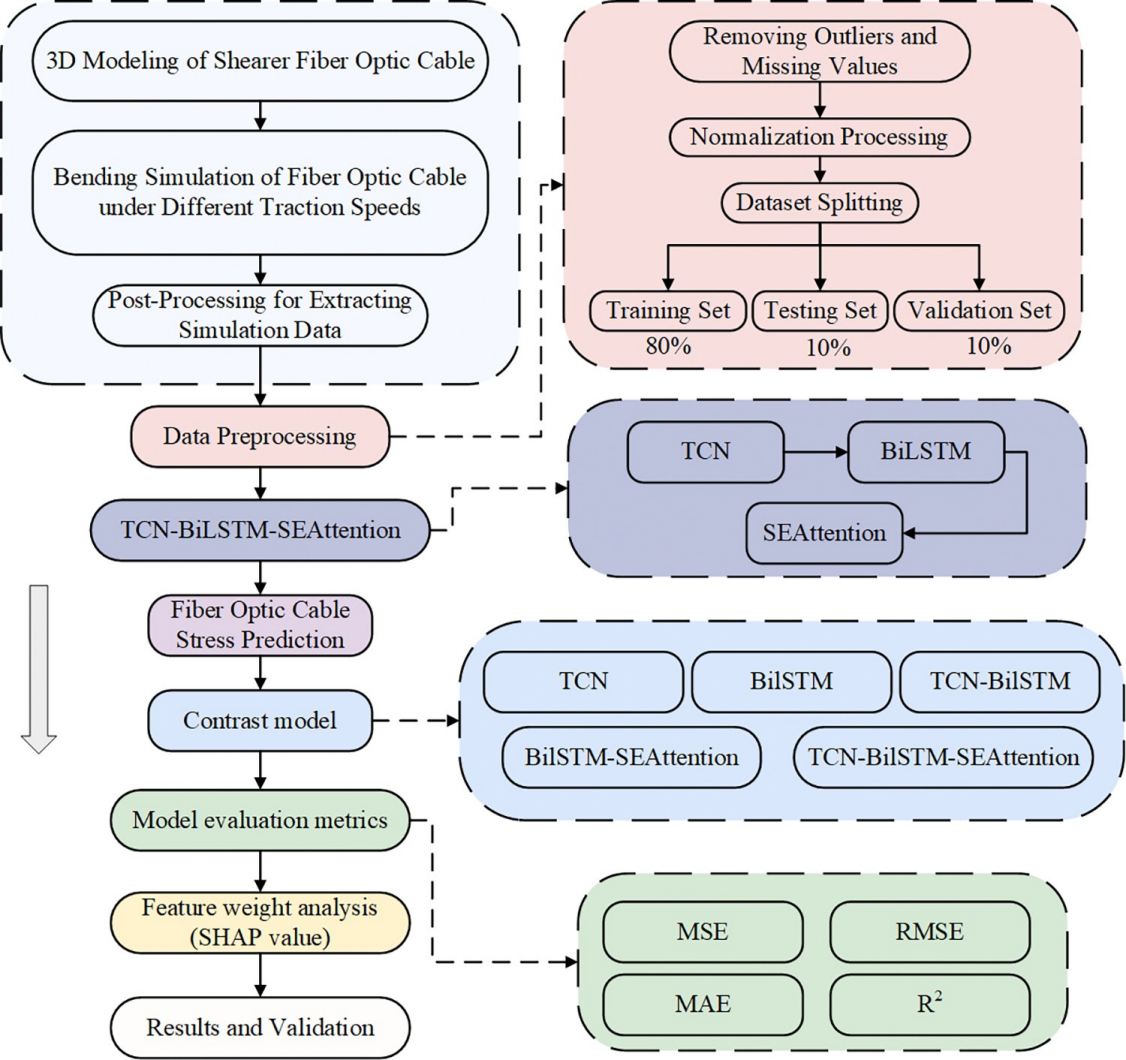
Shearer fiber optic cable stress prediction flowchart.

The above scholars have made significant progress in the structural design, fault diagnosis and prediction of fiber optic cables, but less research has been done on the prediction of mechanical characteristics of shearer fiber optic cables, and the special working conditions and mobile characteristics of shearer fiber optic cables make them the focus of research on intelligent unmanned mining in coal mines. This paper takes the MCPT-1.9/3.3 3*120+1*70+4*10 fiber optic cable as the engineering object, and proposes a method for predicting the mechanical characteristics of shearer fiber optic cable based on TCN-BiLSTM-SEAttention. By learning the change rule of stress between the power conductor, control conductor and optical fiber, it achieves the prediction of the mechanical characteristics of fiber optic cables under bending conditions through optical fiber stress, and verifies the validity of the method through multiple sets of experiments. The Shearer Fiber Optic Cable Stress Prediction Flowchart is shown in Fig 1.

## 2. Methodology

### 2.1 TCN

**Fig 2:**
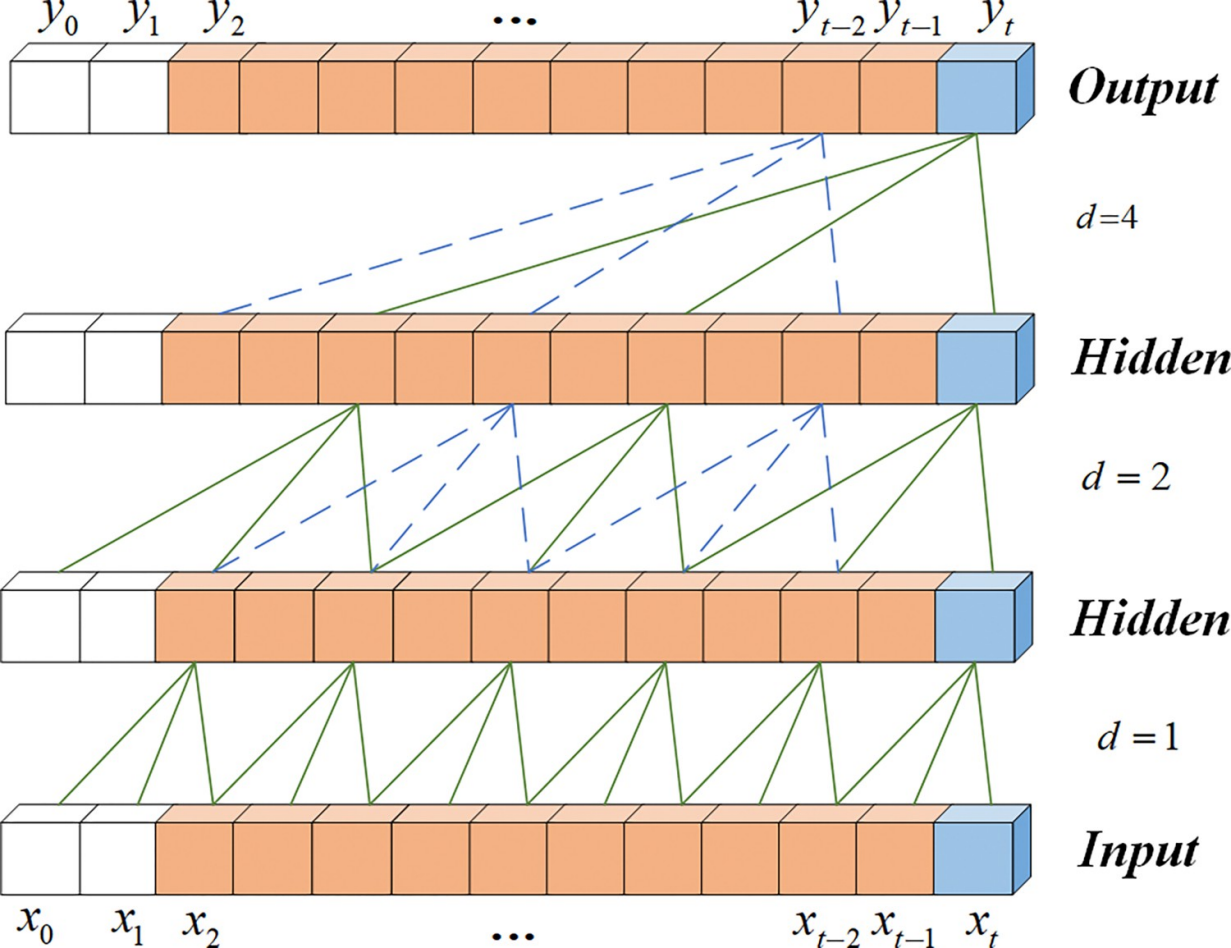
Causal convolution diagram of the TCN network.

TCN is a deep learning model specifically designed for processing time series data [20]. Compared to traditional RNNs and LSTMs, TCN can more efficiently capture long-term dependencies in time series data through convolutional operations. TCN uses one-dimensional causal convolutions, ensuring that the output depends only on the current and previous inputs, thus maintaining the temporal nature of the data. This is especially important for the bending conditions of the optical fiber cable. The stress variations of the cable exhibit significant temporal dependencies, where the stress state at the current moment is determined by the conditions at previous moments, and the state at future moments should not influence the current prediction. Additionally, TCN introduces dilated convolutions, allowing the model to capture long-range temporal dependencies, thereby enhancing the modeling of long-term dependencies. This convolutional operation not only allows for parallel computation, speeding up the training process, but also enables flexible feature extraction across different time scales by adjusting the convolution kernel size and depth. Compared to standard convolutional models, TCN has significant advantages in capturing long-term dependencies and improving computational efficiency. Standard convolutional models have a smaller receptive field and typically require stacking more convolutional layers to expand the receptive field, which increases computational complexity. Additionally, they lack causal constraints, which may prevent them from accurately capturing the temporal dependencies during the bending process of the optical fiber cable, the causal convolution of the TCN network is shown in Fig 2.

**Fig 3:**
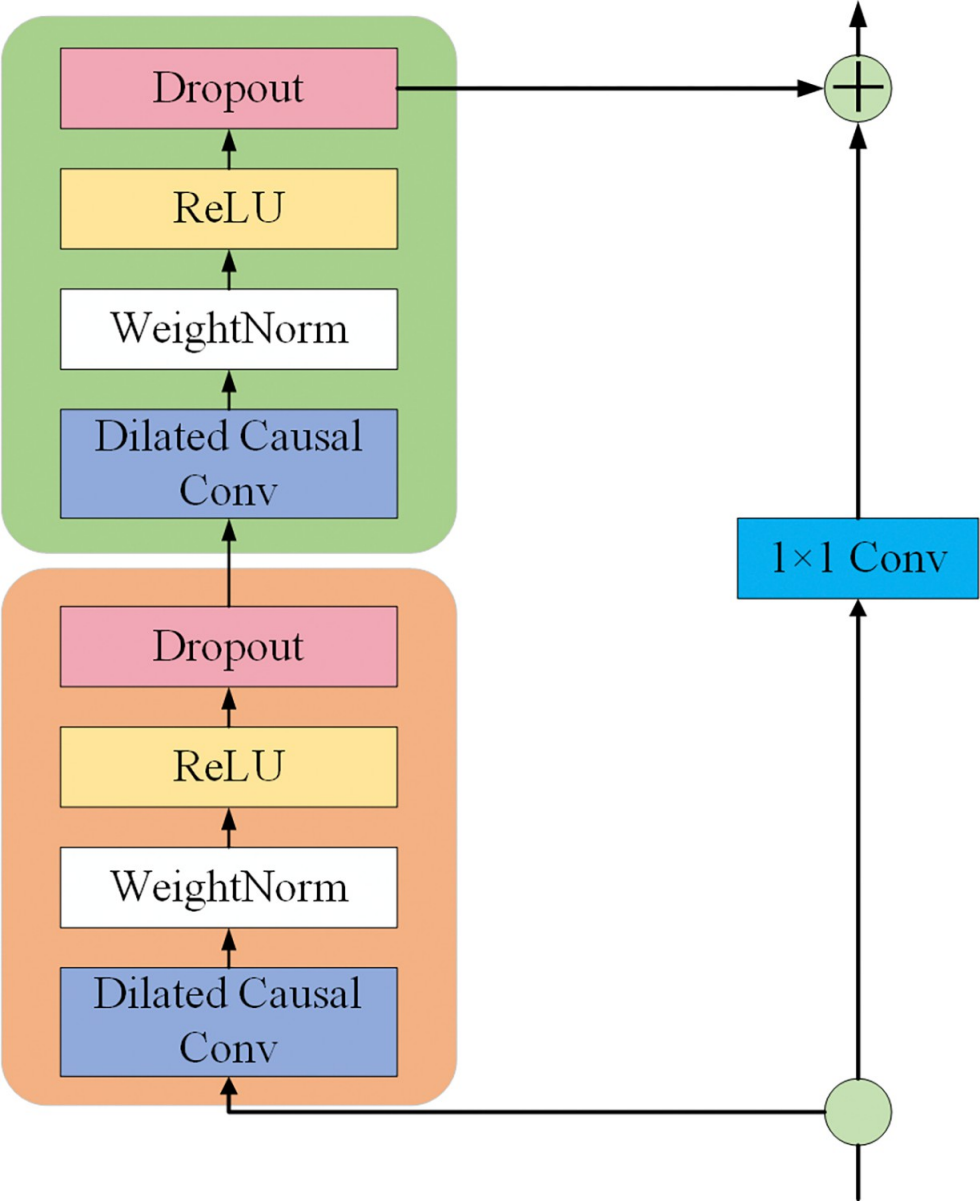
The structure of the TCN network.

To improve training efficiency and stability, TCN employs a residual block structure, where every two convolutional layers are encapsulated in a residual block, avoiding the vanishing gradient problem and promoting smooth information flow. The residual block accelerates training and enhances the model’s representational ability by adding the input signal to the convolutional layer’s output. Stacking multiple residual blocks builds a deep network, with each layer extracting more complex temporal features, thereby improving the model’s accuracy and stability in time series prediction [21, 22]. The structure of the TCN network is illustrated in Fig 3.

### 2.2 BiLSTM

**Fig 4:**
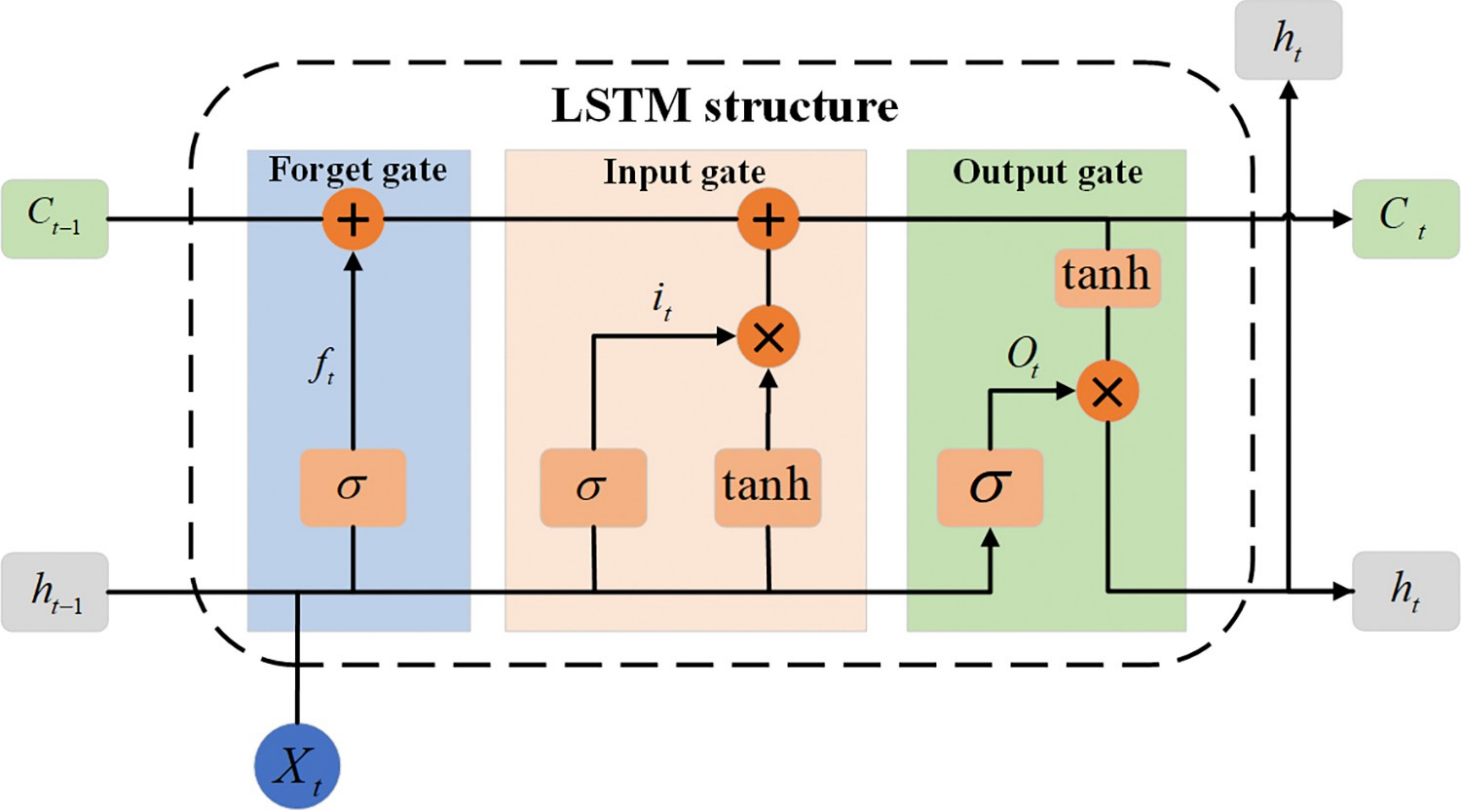
The structure of the LSTM.

LSTM is an improved version of the Recurrent Neural Network (RNN), specifically designed to capture long-term dependencies. Compared to traditional RNNs, LSTM addresses the issues of gradient vanishing and explosion by introducing gating mechanisms, making it more effective in handling long sequence data. LSTM is widely used in fields such as time series forecasting, natural language processing, speech recognition, and machine translation [23]. The structure of the LSTM is shown in Fig 4.

The computation formulas of the LSTM network are as follows:

$$f_{t}=\sigma (W_{f}\cdot [h_{t-1},x_{t}]+b_{f})$$ (1)

$$i_{t}=\sigma (W_{i}\cdot [h_{t-1},x_{t}]+b_{i})$$ (2)

$${\tilde{C}}_{t}=\tanh(W_{C}\cdot [h_{t-1},x_{t}]+b_{C})$$ (3)

$$C_{t}=f_{t}*C_{t-1}+i_{t}*{\tilde{C}}_{t}$$ (4)

$$o_{t}=\sigma (W_{o}\cdot [h_{t-1},x_{t}]+b_{o})$$ (5)

$$h_{t}=o_{t}\cdot \tanh(C_{t})$$ (6)

Here, *f*_*t*_, *i*_*t*_, and *o*_*t*_ represent the forget gate, input gate, and output gate, respectively. *C*_*t*_ denotes the cell state value at time t, while *h*_*t*_ and *h*_*t*−1_ represent the output of the hidden state and the input of the hidden state at time t, respectively. *W*_*f*_, *W*_*i*_, *W*_*C*_, and *W*_*o*_ are the weight matrices, and *b*_*f*_, *b*_*i*_, *b*_*C*_ and *b*_*o*_ are the bias vectors. *σ* and tanh represent the Sigmoid function and the hyperbolic tangent function, respectively.

**Fig 5:**
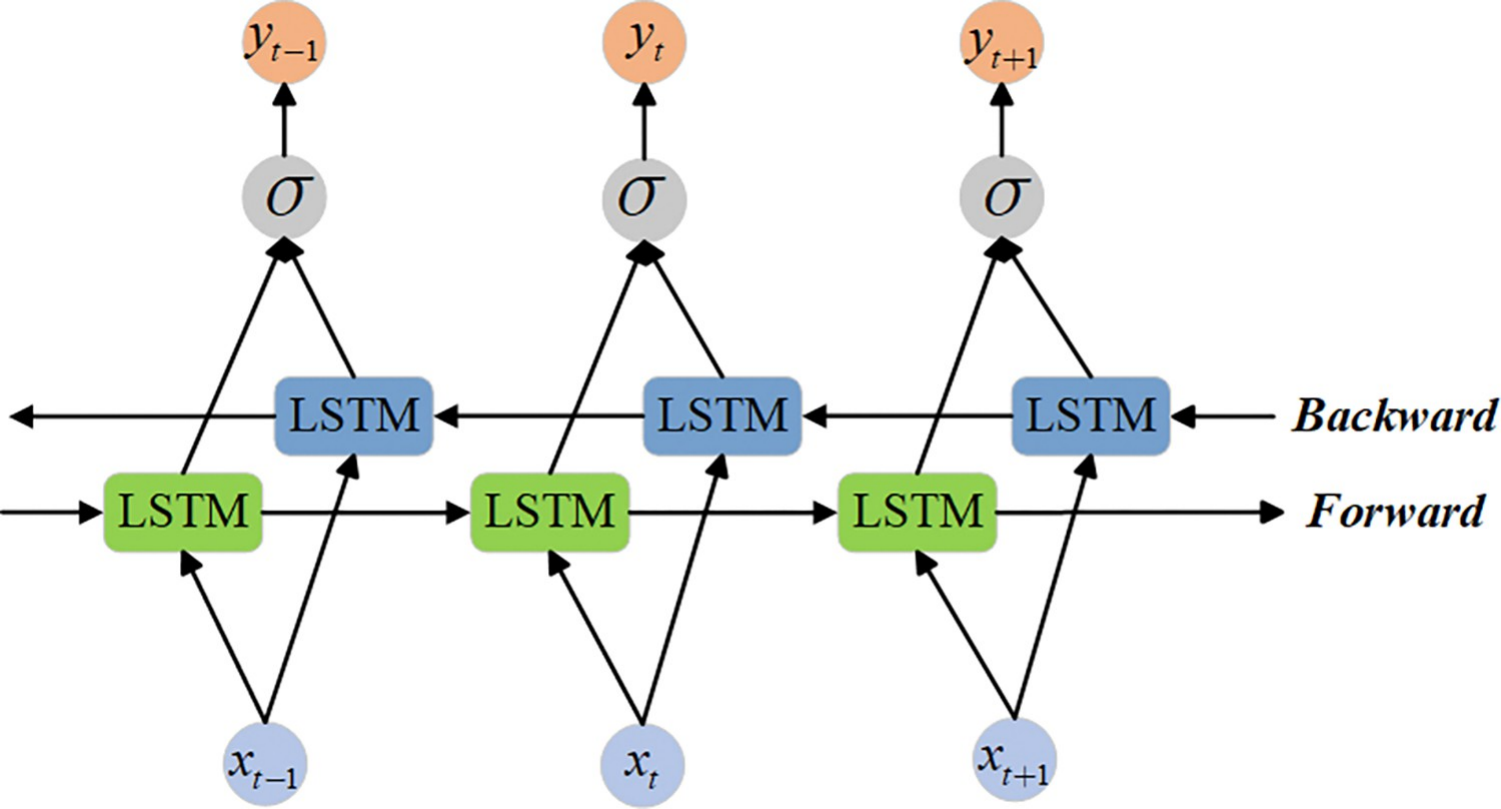
The structure of the BiLSTM.

The BiLSTM [24] consists of two LSTMs, each processing the forward and backward information of the time series, enabling the model to simultaneously capture the contextual information at the current time step and better understand the dynamic changes in the time series data. In traditional LSTM, the model can only make predictions based on past time steps, unable to fully leverage future information. Compared to LSTM, BiLSTM, by sharing information in both forward and backward propagation, enhances the model’s memory capability for the sequence. The structure of the BiLSTM is shown in Fig 5.

### 2.3 SEAttention

In deep learning, the attention mechanism can quickly extract key features from large data, reduce computational costs, and improve learning efficiency and accuracy [25].

**Fig 6:**
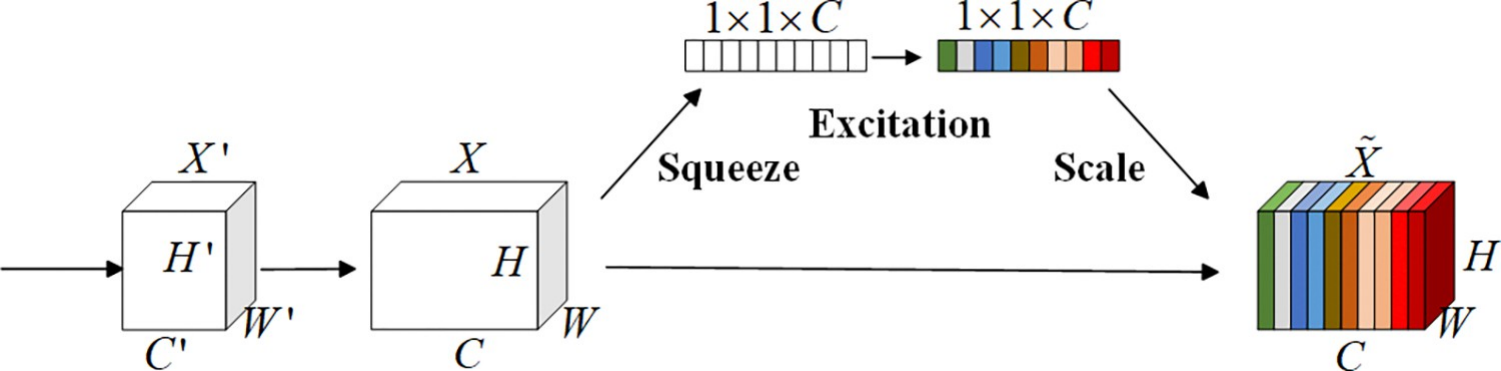
The structure of the SEAttention.

SEAttention enhances the capture of key features by adaptively and dynamically adjusting the weights of the feature channels. The SE modules include Squeeze, Excitation and Scale [26]. The structure of the SEAttention is shown in Fig 6.

As shown in Fig 5, *X*’ represents the original data, while *H*’, *W*’, and *C*’ denote the height, width, and number of channels of the original input, respectively. *X* represents the convolved data, and *H*, *W*, and *C* are the height, width, and number of channels of the convolved data, respectively. $\tilde{X}$ is the feature recalibration result. In the Squeeze stage, the module performs global average pooling to capture global information. During the excitation stage, a multi-layer perceptron (MLP) processes the channel descriptors to generate weights for each channel. Finally, the channel weights are generated and used to weight the BiLSTM output features.

SEAttention effectively reduces the impact of low-variance and noisy features through a weighting mechanism. During training, low-variance features are dynamically assigned lower weights, while the influence of noisy features is suppressed, thereby preventing them from interfering with the model’s predictions. This ensures the robustness and accuracy of the model in real-world applications. Through this mechanism, the model can focus more on the features that have higher predictive value in optical fiber cable bending stress prediction.

## 3. TCN-BiLSTM-SEAttention model

In this paper, TCN and SEAttention are integrated into the BiLSTM model to construct a novel deep learning architecture, TCN-BiLSTM-SEAttention, aimed at improving the predictive capability for the stress of shearer fiber optic cables under bending conditions.

The unique one-dimensional causal convolution structure of TCN ensures the time-series characteristics of shearer fiber optic cable data, while the residual connection unit accelerates network convergence. Additionally, the dilated convolution ensures that the stress features of each core are adequately extracted, which accelerates the training process. As the core component of the model, BiLSTM is capable of utilizing both past and future information, which is critical for stress prediction in fiber optic cables. Through its gating mechanism, BiLSTM effectively retains important stress features and discards irrelevant information, thereby enhancing the prediction accuracy. SEAttention allows the model to automatically focus on key features when processing fiber optic cable data. By calculating the importance weights of these features, the model can sufficiently capture the complex relationships between power conductors, control conductors, and fiber optic stresses, thus improving prediction performance and generating more accurate results.

**Fig 7:**
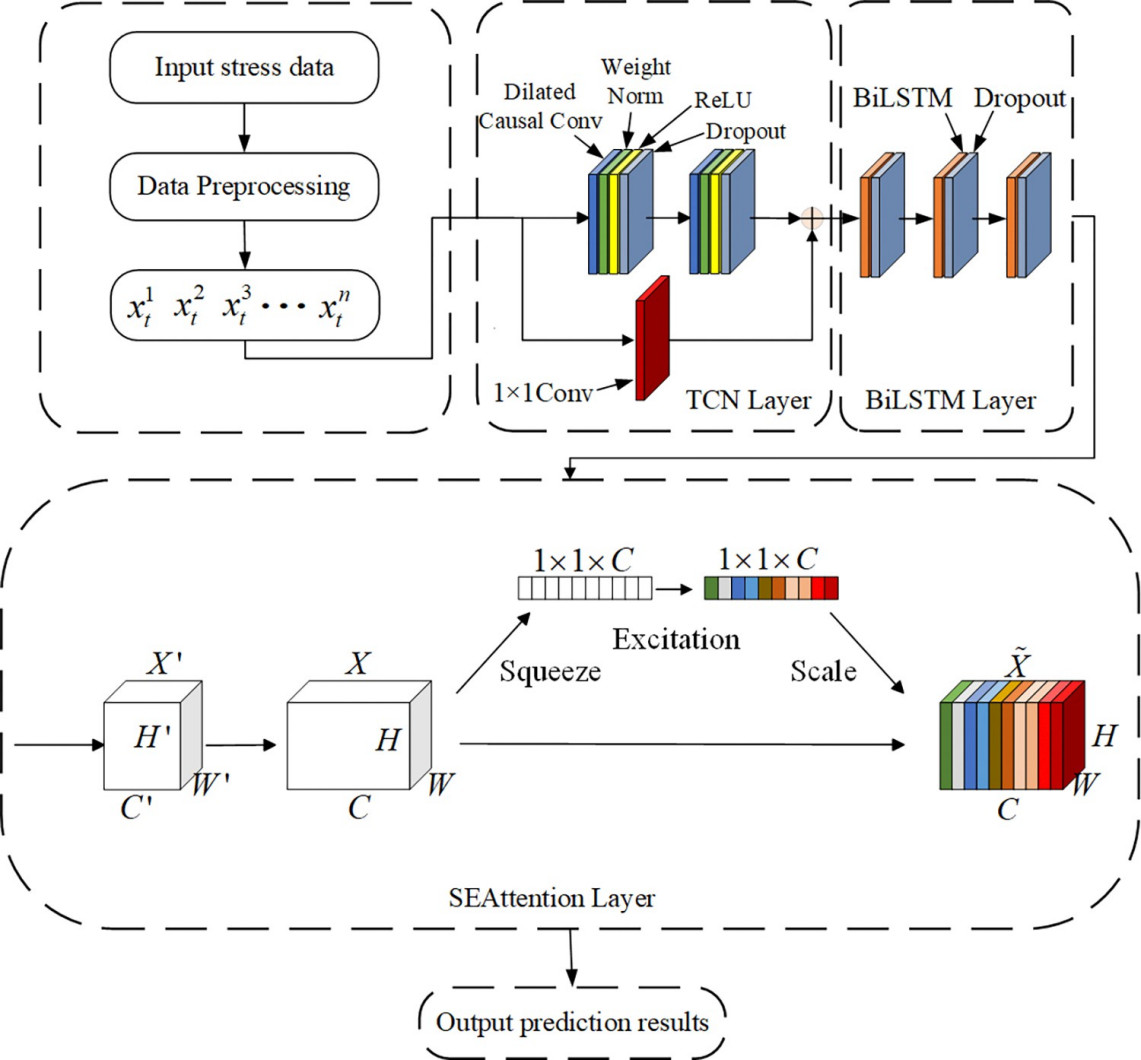
TCN-BiLSTM-SEAttention model framework.

After the adjustment of network parameters, the TCN-BiLSTM-SEAttention prediction model with the best training effect can be obtained, and the model framework is shown in Fig 7.

## 4. Modeling and simulation of shearer fiber optic cables

### 4.1 Parametric modeling of shearer fiber optic cables

**Fig 8:**
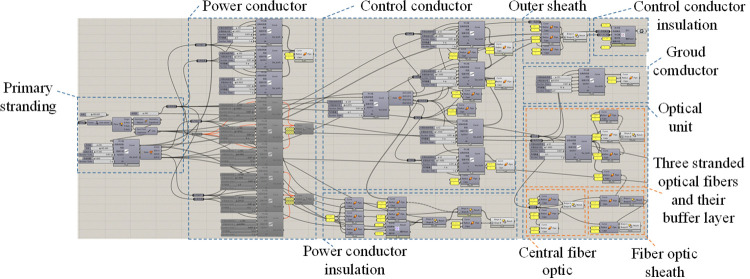
Functional blocks of the shearer fiber optic cable structure. 1-Outer sheath 2-Power conductor insulation 3-Power conductor 4-Ground conductor 5-Control conductor. 6-Control conductor insulation 7-Small sheath 8-Fiber optic sheath 9- Fiber optic 10- Fiber optic buffer layer.

**Fig 9:**
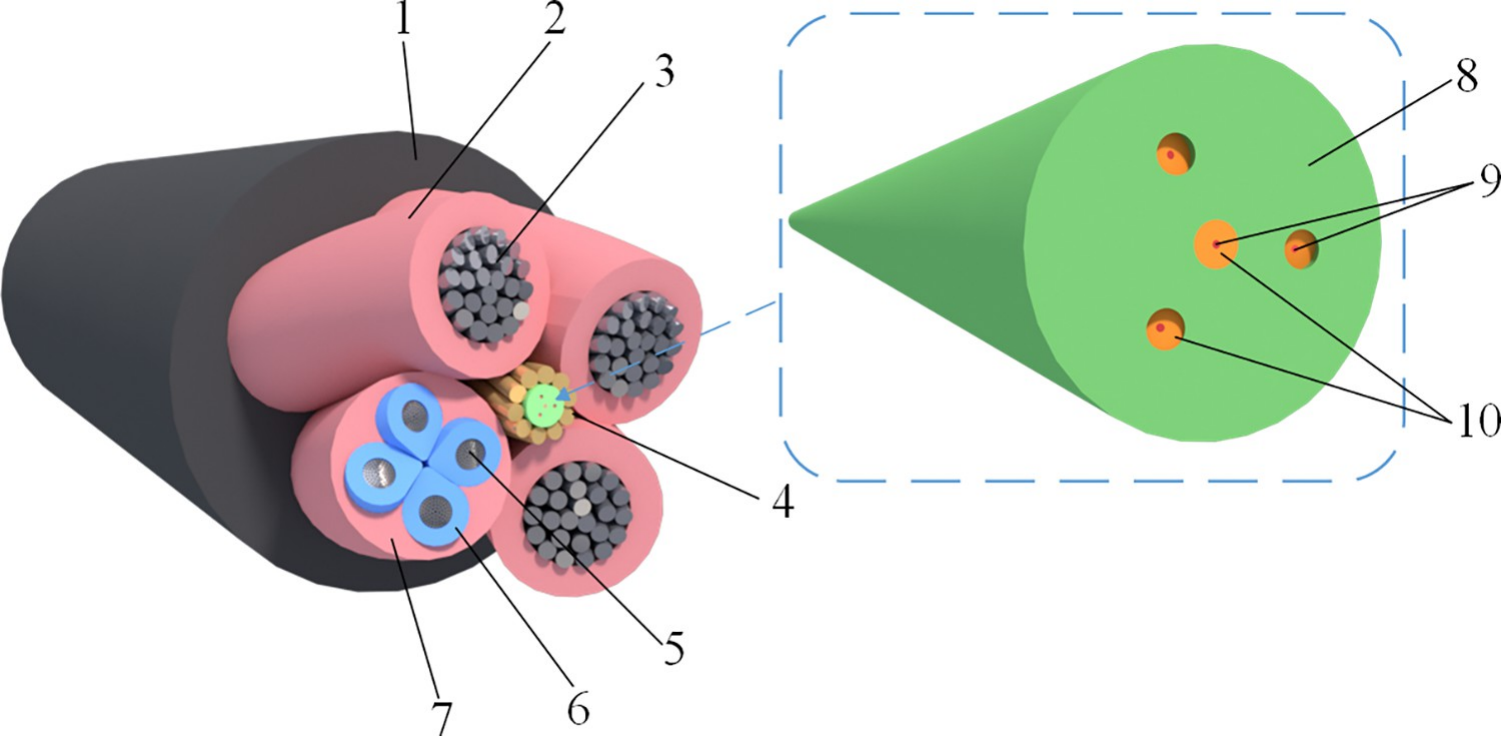
The constructed model of the shearer fiber optic cable.

Parametric modeling was performed using the Grasshopper module and a node-based visual interactive programming method developed by the project team [27]. The functional blocks of the shearer fiber optic cable structure are shown in Fig 8, and the constructed model of the shearer fiber optic cable is shown in Fig 9.

### 4.2 Bending condition simulation of shearer fiber optic cables

**Fig 10:**
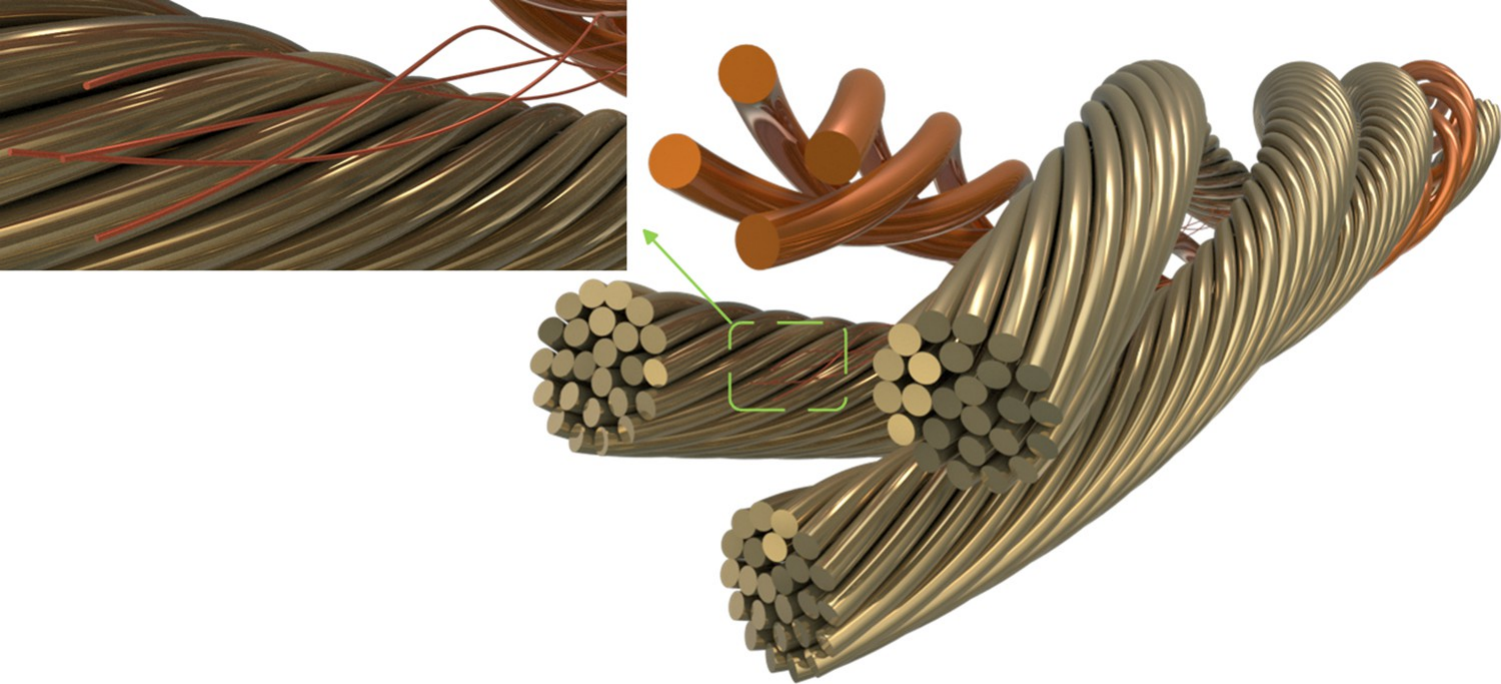
Equivalent simplified model of the fiber optic cable.

**Fig 11:**
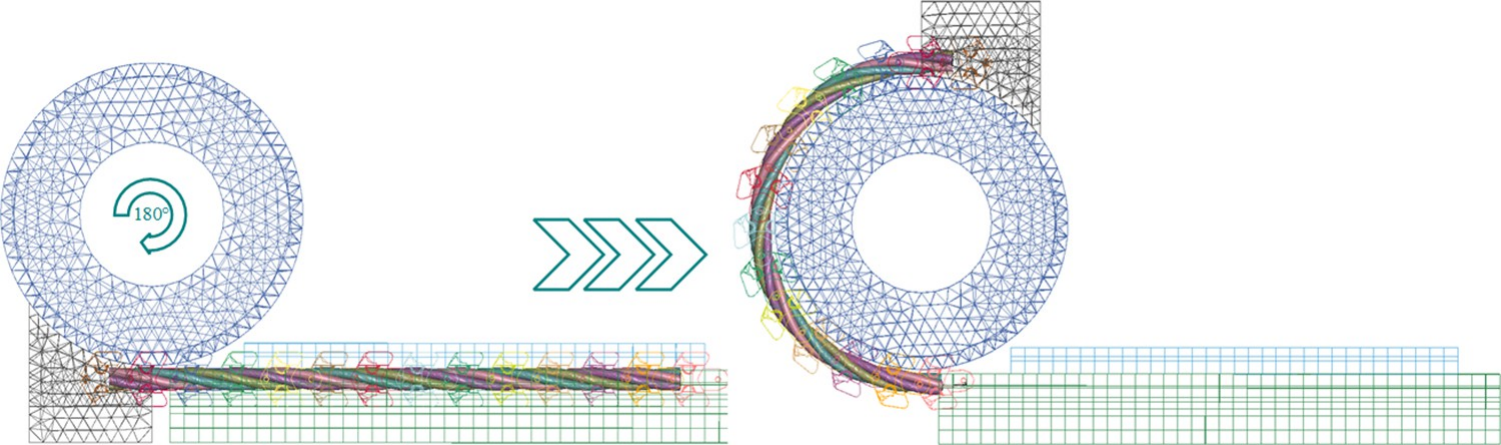
Schematic of fiber optic cable bending simulation model.

The contact relationships among the strands within the conductor of shearer fiber optic cables are highly complex. Performing finite element analysis (FEA) based on actual operating conditions entails significant computational costs and time. Thus, it is crucial to simplify the cable structure effectively. In this study, the intricate strand structure of the fiber optic cable is simplified into an equivalent cross-sectional cylindrical model to reduce computational complexity and enhance analysis efficiency [28]. The equivalent simplified model of the fiber optic cable is shown in Fig 10. Multiple bending simulations of fiber optic cables were conducted using the Workbench LS-DYNA software [29], and the simulation model is illustrated in Fig 11.

**Fig 12:**
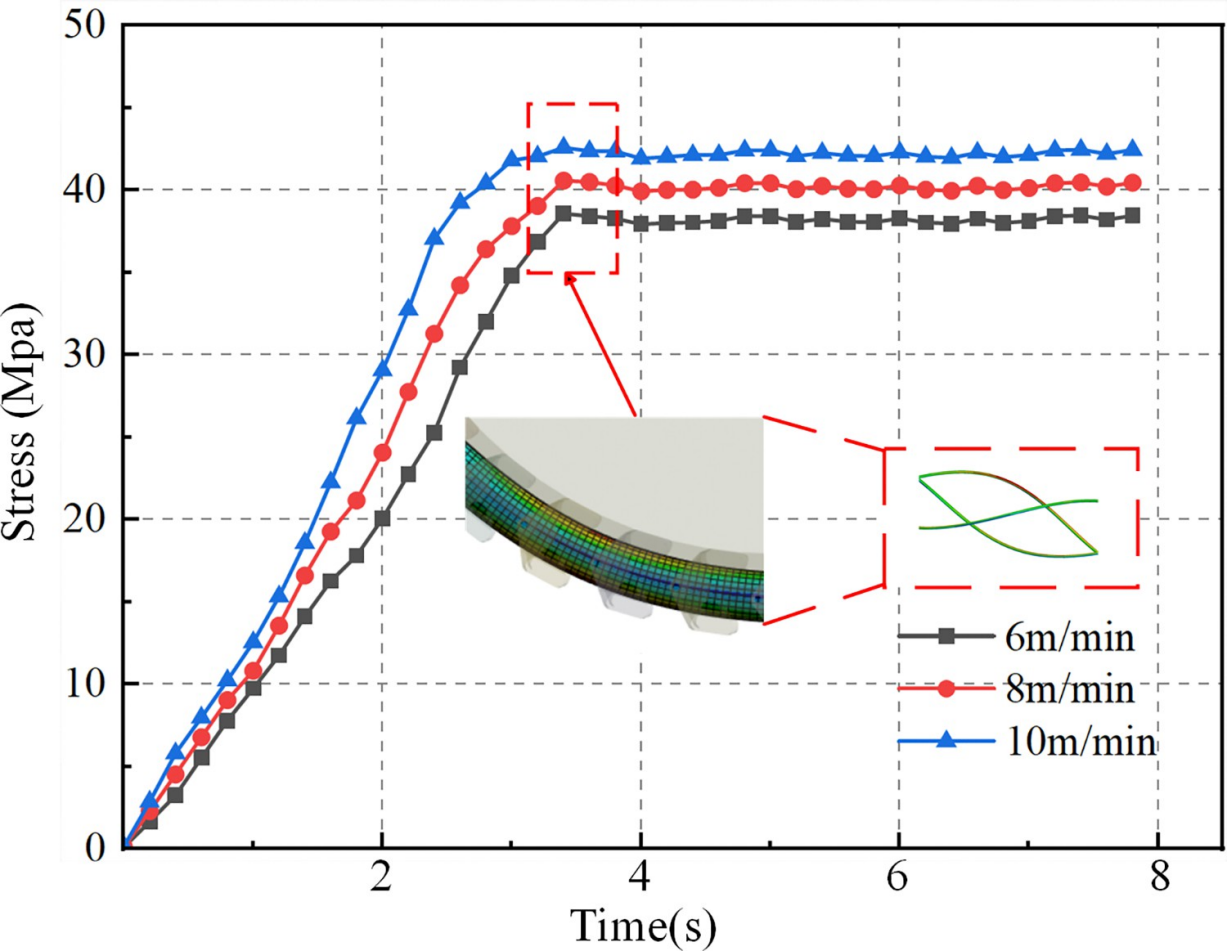
Stress-time variation curve of optical fiber.

The experimental conditions and structural parameters in this study are based on the research of Xia et al. [30]. Set the fiber optic cable length to 1600mm, control unit into a cable pitch ratio of 6, the cable and cable clamp assembly, traction block connected to the cable and cable clamp, around the roller simulation of coal mining machine respectively with the actual operating speed of 6m/min, 8m/min and 10m/min uniform rotation, the angle of 0° to 180° to simulate the cable bending the state of the movement. The conductor and conductor, conductor and insulation are in frictional contact, the static friction coefficient is 0.4, and the dynamic friction coefficient is 0.3. The material optimization is based on the research of H. Adin et al. [31, 32] on the performance optimization of composite materials. By optimizing the structure design and processing techniques of reinforcing materials, they can effectively improve the mechanical properties of composite materials, thereby enhancing the overall performance of the materials. The material selection is based on the research of Bejaxhin, A.B.H. team [33, 34] on the mechanical properties of composite materials. This team conducted experimental and numerical simulation analyses to explore the impact of composite material composition and structural design on mechanical properties, significantly improving the tensile strength, bending stiffness, and impact resistance of the composite materials. Considering the working environment of the shearer optical fiber cable, suitable composite materials and reinforcement methods are chosen in combination with the findings of the aforementioned studies to improve the stability and reliability of the optical fiber cable under complex working conditions. The structural materials and parameters of the shearer fiber optic cable are listed in Table 1. The maximum stress of the optical fiber in the cable was extracted, and the stress variation curve over time is shown in Fig 12.

**Table 1:** Structural materials and parameters of the shearer optical fiber cable.

Structure	Material	Density (kg/m^3^)	Elastic Modulus (GPa)	Poisson’s Ratio	Yield Strength (MPa)
Power conductor	Tinned Copper	6176.6	16.649	0.34	241.492
Power conductor insulation	EPDM	1250	7.08	0.49	——
Control conductor	Tinned Copper	8300	22.873	0.34	185.7
Control conductor insulation	EPDM	1250	7.08	0.49	——
Small sheath	EPDM	1250	7.08	0.49	——
Outer sheath	Neoprene	1550	5.54	0.49	——
Fiber optic	Silica Glass	2203	73.1	0.17	289.2
Fiber optic buffer layer	Polyimide	1350	2.75	0.35	——
Fiber optic sheath	Neoprene	1550	5.54	0.49	——

As shown in Fig 12, when the shearer traction speeds are 6 m/min, 8 m/min, and 10 m/min, the maximum stresses of the optical fiber are 38.6 MPa, 40.6 MPa, and 43.1 MPa, respectively. This indicates that as the bending speed of the fiber optic cable increases, the stress also increases. When the cable reaches the specified bending radius, the stress on the optical fiber stabilizes and eventually reaches a steady-state value.

## 5. Experimental results and analysis

To validate the effectiveness of the proposed model, simulation data capturing the time-varying maximum stress of power conductors, control conductors, and optical fibers under different traction speeds were selected as the dataset. A total of 9 data groups were collected at three different speeds, with each group containing 2000 samples, resulting in a total of 18,000 samples. The dataset was divided into training, validation, and testing sets in a ratio of 80%, 10%, and 10% [35], respectively, to ensure the objectivity and comprehensiveness of the model evaluation. To more comprehensively evaluate the model’s stability and generalization ability, we also conducted a 10-fold cross-validation experiment [36, 37] and compared it with the dataset splitting method. In cross-validation, the data is randomly divided into 10 subsets, and the model undergoes 10 rounds of training and validation, with a different subset used as the validation set in each round, and the remaining subsets used for training.

### 5.1 Data preprocessing

To improve the training efficiency and prediction accuracy of the model, the data was preprocessed before being fed into the model. First, outliers were detected and removed, and missing values were addressed. Second, to prevent dimensional differences from causing prediction errors, the data was normalized before being input into the network:

$$x_{0}=\frac{x-x_{min}}{x_{max}-x_{min}}$$ (7)

Here, *x* represents the original feature data, *x*_*min*_ and *x*_*max*_ are the minimum and maximum values, respectively, and *x*_0_ is the normalized data.

### 5.2 Model evaluation metrics

To evaluate the predictive performance of the model, four evaluation metrics were selected: Mean Squared Error (MSE), Root Mean Squared Error (RMSE), Mean Absolute Error (MAE), and Coefficient of Determination (R^2^) [38]. The definitions and evaluation criteria of each metric are shown in Table 2 and the formulas are as follows:

$$MSE=\frac{1}{n}\sum_{i=1}^{n}{\left({\hat{y}}_{i}-y_{i}\right)}^{2}$$ (8)

$$RMSE=\sqrt{\frac{1}{n}\sum_{i=1}^{n}{\left({\hat{y}}_{i}-y_{i}\right)}^{2}}$$ (9)

$$MAE=\frac{1}{n}\sum_{i=1}^{n}|({\hat{y}}_{i}-y_{i})|$$ (10)

$$R^{2}=1-\frac{\sum_{i=1}^{n}{\left({\hat{y}}_{i}-y_{i}\right)}^{2}}{\sum_{i=1}^{n}{\left({\bar{y}}_{i}-y_{i}\right)}^{2}}$$ (11)

Here, *n* represents the total number of samples, ${\hat{y}}_{i}$ denotes the actual value, *y*_*i*_ is the predicted value, and $\bar{y_{i}}$ is the mean of the actual values.

**Table 2:** The definitions and evaluation criteria of each metric.

Metric	Definition	Evaluation Criteria
MSE	The average of the squared differences between predicted values and actual values.	The closer the value is to 0, the smaller the model’s prediction error, and the better the model performance.
RMSE	The square root of MSE, measuring the standard deviation of prediction errors.	The closer the value is to 0, the higher the model’s prediction accuracy and the closer it is to the actual values.
MAE	The average of the absolute differences between predicted values and actual values.	The closer the value is to 0, the smaller the model’s deviation from the true values, indicating higher prediction accuracy.
R^2^	A measure of the goodness of fit of the model, indicating the proportion of variance explained by the model.	The closer the value is to 1, the better the model fits the data and the stronger its predictive power

### 5.3 Model parameter settings

The experiments were conducted using the PyTorch framework, with an Intel i5-12600KF CPU, an NVIDIA GeForce GTX 4060 GPU, and 32GB of RAM. The system utilized CUDA 10.1, and third-party libraries such as matplotlib, NumPy, Pandas, and sklearn were installed.

The hyperparameters used in the experiments were determined based on relevant literature [39, 40], practical experience, and multiple experimental trials along with model performance evaluations. The specific settings are as follows: Adam optimizer, ReLU activation function, an initial learning rate of 0.001, 100 training iterations, and a batch size of 64. The choice of optimizer and activation function is based on common practices in time-series data prediction, effectively improving the model’s training efficiency and prediction performance. The learning rate, number of iterations, and batch size were fine-tuned through several experiments and adjusted based on practical experience to ensure efficient training and high prediction accuracy. Additionally, Dropout regularization and L2 regularization were applied to prevent overfitting and avoid over-reliance on noisy features during feature selection. These measures enhanced the model’s robustness, ensuring it maintains high prediction accuracy when handling complex shearer optical fiber cable data. The model structure parameters are listed in Table 3.

**Table 3:** Model structure parameters.

Model Structure	Model Parameter	Value
TCN	Convolution kernels	64
	Kernel size	3
	Dilation rate	1,2,4
	Dropout rate	0.3
BiLSTM	Hidden Layer Dimension	128
	Number of Layers	3
SEAttention	Hidden Layer Dimension	256

### 5.4 Results and analysis

**Fig 13:**
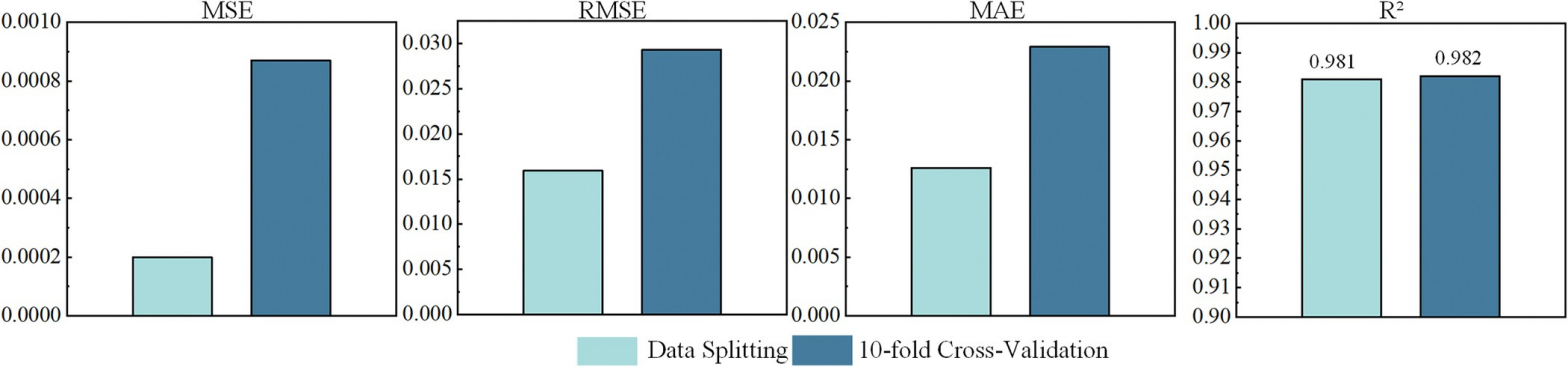
Evaluation metrics of the dataset splitting method and 10-fold cross-validation method.

To assess the stability and generalization ability of the model, the dataset splitting method was compared with the 10-fold cross-validation method. The comparison of the two methods across various evaluation metrics is shown in Fig 13.

As shown in Fig 13, the average MSE for the cross-validation method is 0.000868, RMSE is 0.0293, MAE is 0.0229, and R^2^ is 0.982. The MSE for the dataset splitting method is 0.00020, RMSE is 0.0159, MAE is 0.0126, and R^2^ is 0.981. The results indicate that while the cross-validation method has slightly higher errors, the R^2^ values of both methods are very close, suggesting similar prediction capabilities. Therefore, the computational efficiency of the model is particularly important in this study. Compared to cross-validation, the dataset splitting method significantly reduces computation time while maintaining high prediction accuracy. Based on the computational efficiency and data volume of the experiments, the dataset splitting method was ultimately chosen for subsequent experiments.

**Fig 14:**
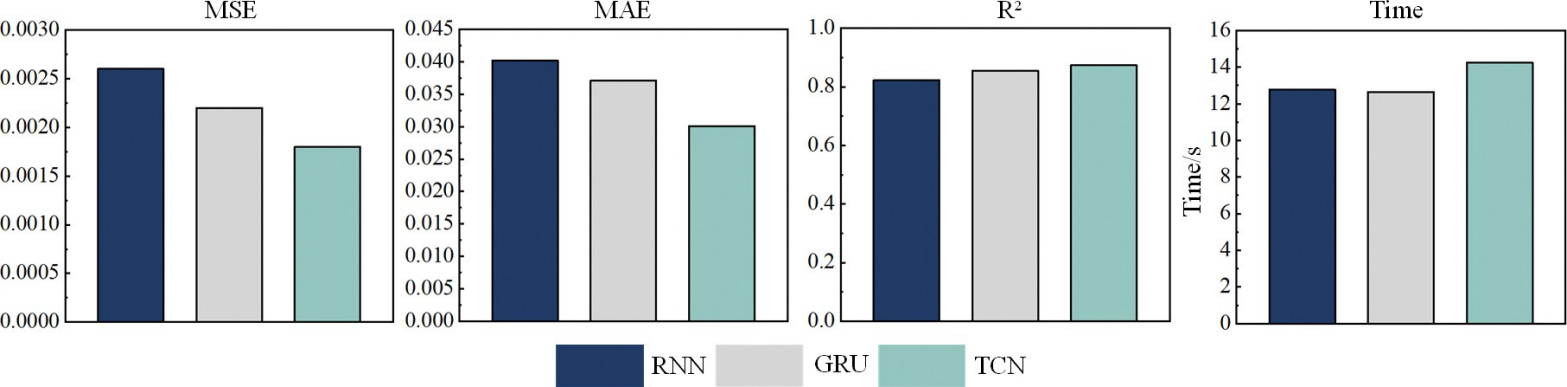
Evaluation metrics for TCN, RNN, and GRU models.

This study compares several common time-series models, including TCN, RNN, and GRU, to evaluate their performance in predicting bending stress in shearer optical fiber cables. The evaluation metrics for each model are shown in Table 4. Fig 14 presents a histogram of the evaluation metrics for each model.

**Table 4:** Evaluation metrics for TCN, RNN, and GRU models.

Model	MSE	MAE	R^2^	Times/s
RNN	0.0026	0.0402	0.822	12.78
GRU	0.0022	0.0371	0.854	12.65
TCN	0.0018	0.0301	0.873	14.26

As shown in Table 4 and Fig 14, the computation times for GRU and RNN are relatively short, at 12.65s and 12.78s, respectively, while the computation time for TCN is slightly longer at 14.26s. However, compared to the classic sequential models RNN and GRU, TCN achieves a reduction in MSE by 0.0008 and 0.0004, a reduction in MAE by 0.0101 and 0.007, and an improvement in R^2^ by 0.051 and 0.019, respectively. TCN outperforms in all performance metrics, demonstrating higher prediction accuracy. Specifically, in predicting the bending stress of coal mine optical fiber cables, where the stress exhibits significant periodicity, TCN, with its causal convolution and dilated convolution mechanisms, effectively captures this periodic feature. Therefore, despite the slightly longer computation time, TCN’s higher prediction accuracy makes it more advantageous in practical applications, especially when precise modeling of long-term dependencies and complex patterns is required. Based on the above comparative analysis, TCN is selected as the baseline model for subsequent experiments.

**Fig 15:**
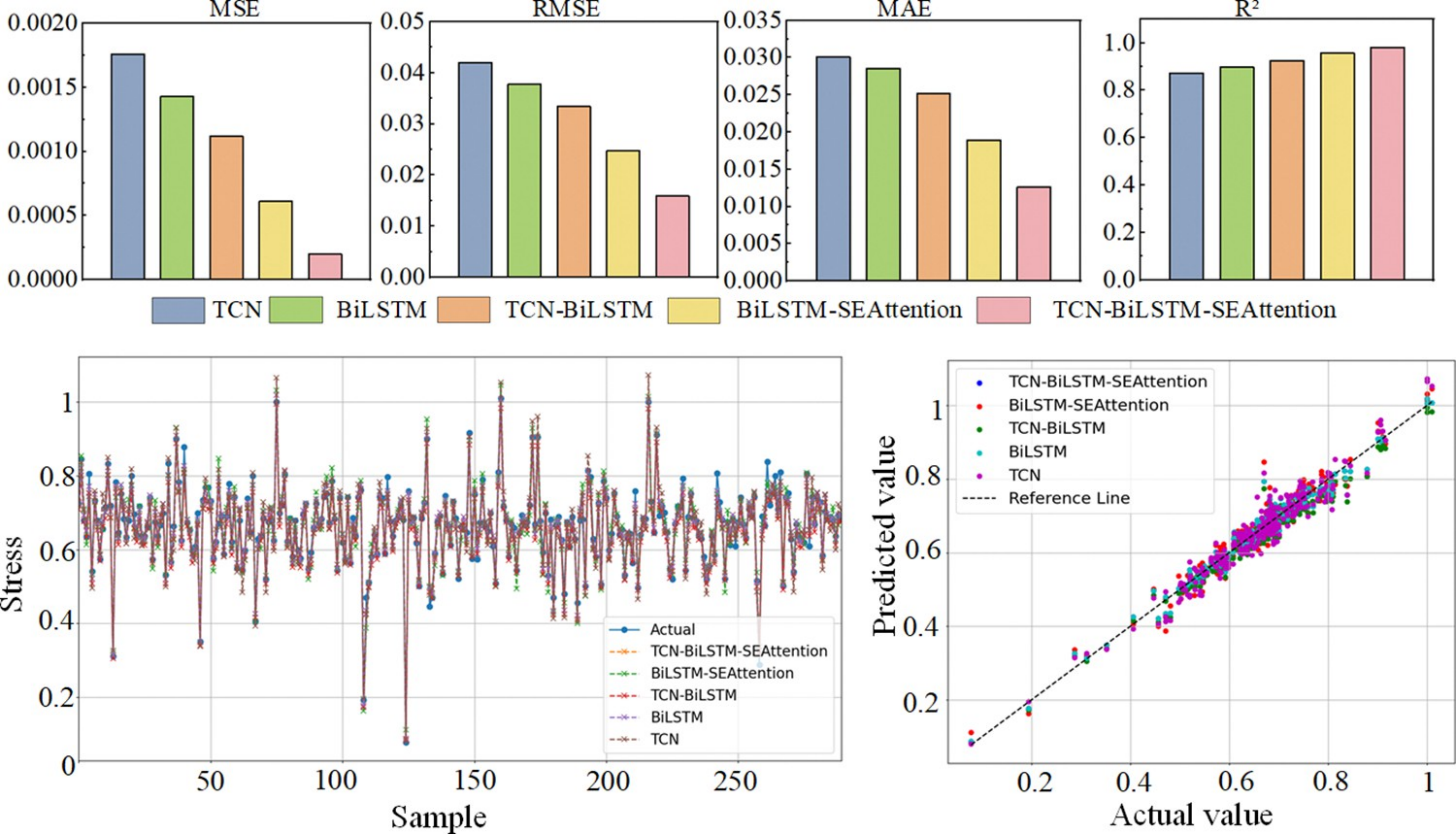
Prediction results and evaluation metrics of each model.

Comparative analysis was performed using individual models of TCN, BiLSTM, and the combined models of TCN-BiLSTM, BiLSTM-SEAttention, and TCN-BiLSTM-Attention. Mechanical performance was selected as the evaluation metric to assess and quantify the models, enabling the prediction of the mechanical properties of shearer fiber optic cables during bending. The evaluation metrics of each model are shown in Table 5. The prediction results and evaluation metrics for each model are shown in Fig 15.

**Table 5:** Evaluation metrics of each model.

Model	MSE	RMSE	MAE	R^2^
TCN	0.00176	0.0419	0.0301	0.873
BiLSTM	0.00143	0.0378	0.0285	0.897
TCN-BiLSTM	0.00112	0.0334	0.0252	0.925
BiLSTM-SEAttention	0.00061	0.0247	0.0189	0.956
TCN-BiLSTM-Attention	0.00050	0.0230	0.0189	0.961
TCN-BiLSTM-SEAttention	0.00020	0.0159	0.0126	0.981

As shown in Fig 15 and Table 5, the proposed model in this study demonstrates outstanding performance in predicting the mechanical properties of shearer optical fiber cables, outperforming other models in all evaluation metrics. Specifically, the model achieves an R^2^ of 0.981, MSE of 0.00020, RMSE of 0.0159, and MAE of 0.0126. Compared to the individual TCN and BiLSTM models, the proposed model reduces MSE by 0.00156 and 0.00123, RMSE by 0.026 and 0.0219, MAE by 0.0175 and 0.0159, and improves R^2^ by 0.108 and 0.084, respectively. This improvement may be due to the limitations of the individual TCN and BiLSTM models in capturing long-range dependencies and handling complex feature relationships. When comparing the combined models of TCN-BiLSTM and BiLSTM-SEAttention, the proposed model reduces MSE by 0.00092 and 0.00041, RMSE by 0.0175 and 0.0088, MAE by 0.0126 and 0.0063, and improves R^2^ by 0.056 and 0.025, respectively. The reason for these differences may lie in the limitations of the TCN-BiLSTM model in feature extraction and attention mechanism, while the BiLSTM-SEAttention model is less effective in handling model depth and complexity compared to the proposed model.

In conclusion, the proposed model combines the strengths of TCN and BiLSTM and further enhances the ability to capture key features in the data through the SEAttention mechanism, which results in superior prediction accuracy and data fitting performance, demonstrating its excellence in predicting the mechanical properties of shearer optical fiber cables under bending conditions.

**Fig 16:**
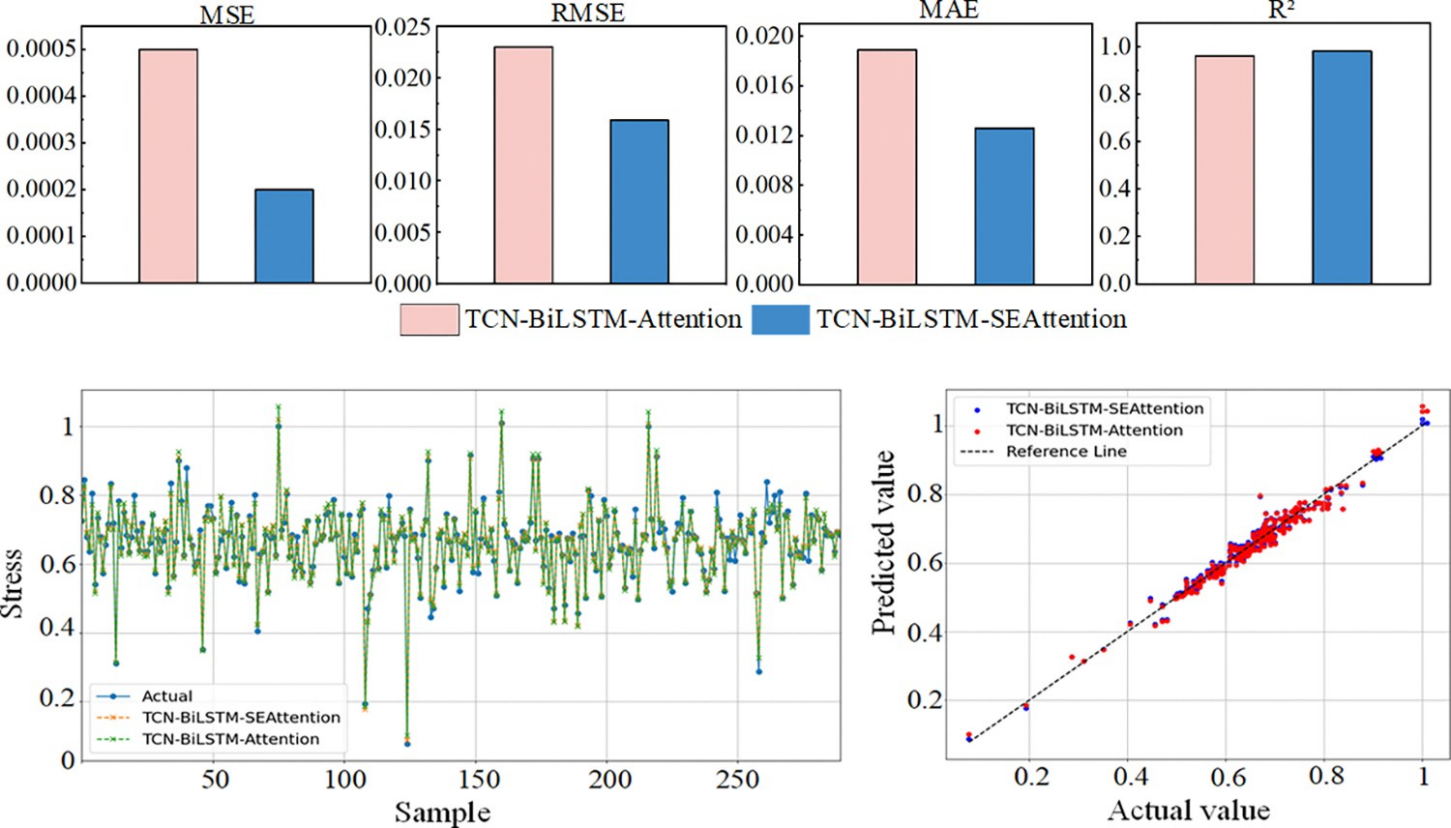
Prediction results and evaluation metrics of each model.

To further validate the performance advantages of the proposed model, a comparative experiment was conducted with the TCN-BiLSTM-Attention model. The experiment aims to verify whether the SE module can improve the model’s ability to select key features. Therefore, this study compares the performance of the two models on the same dataset and analyzes in detail the enhancement effect of incorporating the SE module on the model’s prediction accuracy and feature selection capability. The prediction results and evaluation metrics for each model are shown in Fig 16.

As shown in Fig 16, compared to the TCN-BiLSTM-Attention model, the proposed model achieves an MSE of only 0.0002, with reductions of 0.0071 and 0.0063 in RMSE and MAE, respectively. The R^2^ reaches 0.981, demonstrating a significant advantage in prediction accuracy.

The performance improvement may be attributed to the structural optimization of the TCN-BiLSTM-SEAttention model. By introducing the SE module, the model’s ability to capture and select key temporal features is further enhanced, allowing for more accurate relationships between the power conductors, control conductors, and optical fiber stress. The SE module adaptively adjusts the weight of each feature, improving the model’s performance in shearer optical fiber cable stress prediction. This optimization makes the model more accurate in predicting stress under the bending conditions of optical fiber cables.

**Fig 17:**
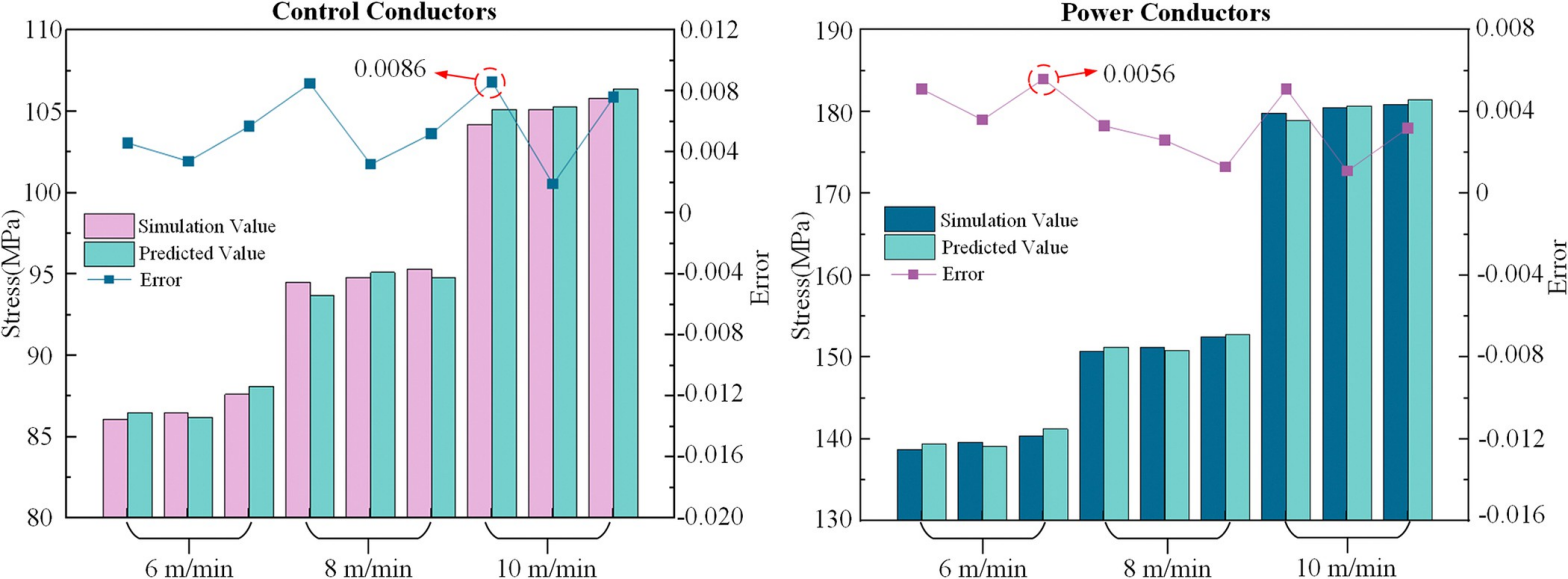
Comparison of simulated and predicted values of power and control conductors at different speeds and the error.

In this study, several representative experimental datasets were selected, using optical fiber stress data as input to predict the stress in the power conductors and control conductors. The prediction results were compared with simulation values to further validate the model’s prediction accuracy. The comparison of simulated and predicted values of power and control conductors, along with the error, is shown in Table 6 and Fig 17.

**Table 6:** Comparison of predicted values and actual values.

Traction Speed (m/min)	Fiber Optic Stress (MPa)	Power Conductor Stress (MPa)		Control Conductor Stress (MPa)		Error between simulation values and predicted values	
		Simulation Value	Predicted Value	Simulation Value	Predicted Value	Power Conductor	Control Conductor
6	37.5	138.7	139.4	86.1	86.5	0.51%	0.46%
	38.1	139.6	139.1	86.5	86.2	0.36%	0.34%
	38.6	140.4	141.2	87.6	88.1	0.56%	0.57%
8	39.5	150.7	151.2	94.5	93.7	0.33%	0.85%
	40.1	151.2	150.8	94.8	95.1	0.26%	0.32%
	40.6	152.5	152.7	95.3	94.8	0.13%	0.52%
10	42.7	179.8	178.9	104.2	105.1	0.51%	0.86%
	43.1	180.5	180.7	105.1	105.3	0.11%	0.19%
	42.3	180.9	181.5	105.8	106.4	0.32%	0.76%

As shown in Table 6 and Fig 17, the maximum prediction error for the power conductors is 0.56%, and for the control conductors, it is 0.86%. This indicates that using optical fiber stress data as input can effectively predict the stress variation in both the power conductors and control conductors, with the model providing accurate predictions under different conditions.

In summary, the TCN-BiLSTM-SEAttention model, using power conductors, control conductors, and optical fiber stress data as input features, demonstrates excellent performance in both accuracy and stability, as evidenced by its high R^2^ value and low error metrics. It offers a more reliable solution for shearer cable stress monitoring and performance prediction. These results provide strong support for future research and practical applications, highlighting the importance and potential of the SEAttention mechanism in enhancing model performance. The findings indicate that this model will have significant implications for future industrial monitoring and prediction.

### 5.5 Feature weight analysis

To improve the accuracy of mechanical property prediction for shearer fiber optic cables during bending and to enhance the interpretability of the model, this study introduces SHAP theory [41]. A thorough analysis of SHAP values provides a precise quantification of the feature contribution to the mechanical property prediction of the fiber optic cables, thereby identifying the most significant features affecting their mechanical performance. For any given sample, the Shapley value of the feature parameter *i* is determined by the following formula:

$$Shapley(i)=\sum_{S\subseteq N\backslash \{i\}}\frac{|S|!(n-|S|-1)!}{n!}[f(S\cup \{i\})-f(S)]$$ (12)

Here, *N* represents the set of all feature parameters, with a total of *n* dimensions. *S* denotes a selected feature subset from *N*, and *f*(*S*) represents the model prediction obtained from training on the feature subset. *f*(*S*∪{*i*}) represents the model prediction obtained from training on the feature subset *S* and the full feature set *N*.

The SHAP value is used as an evaluation metric, where a higher value indicates a greater impact of the feature on the fiber optic cable [42]. The combination of this predictive model with SHAP values enhances the transparency of the model, providing strong data support for subsequent model optimization and improvement. It deepens the understanding of how different features influence the model’s prediction results.

**Fig 18:**
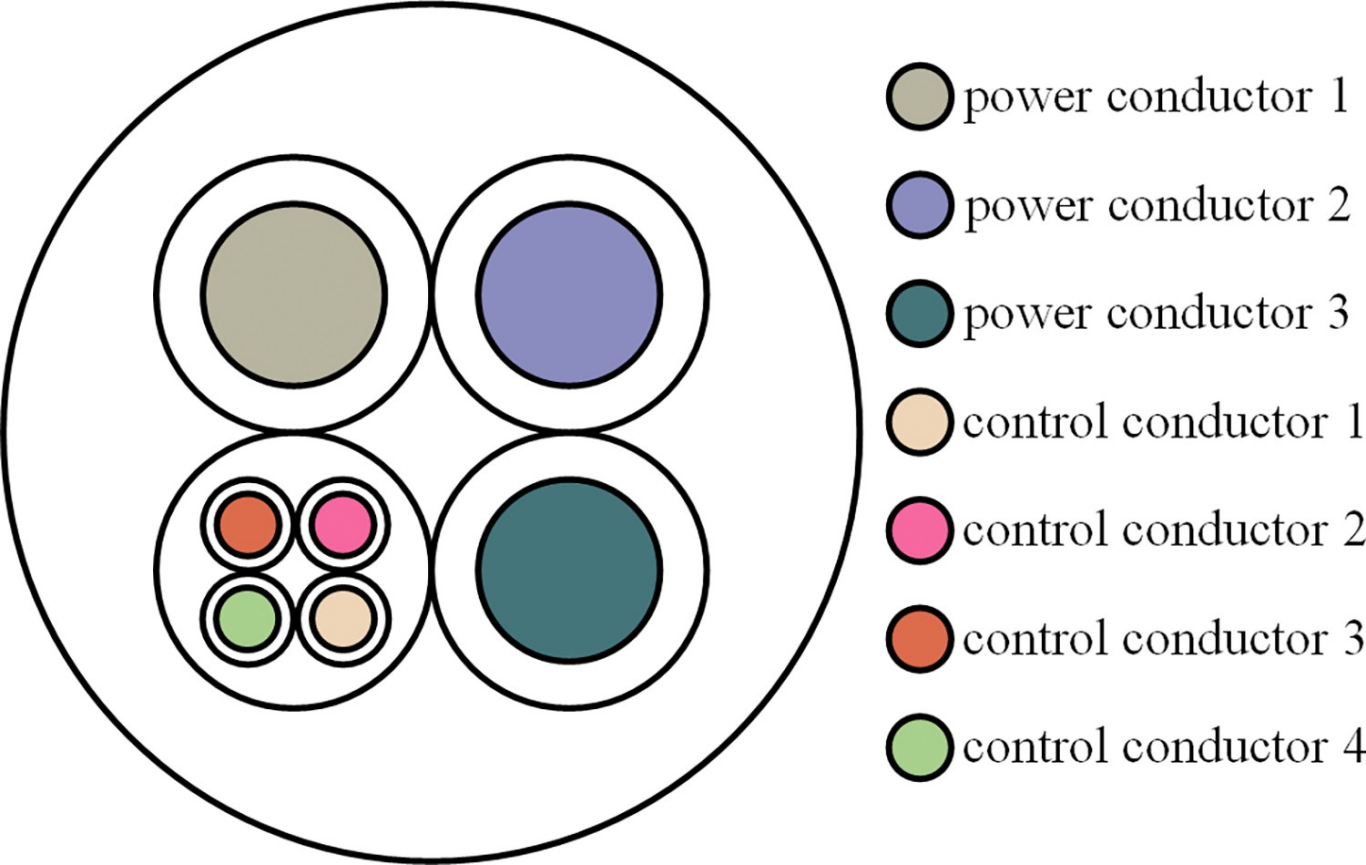
Separation diagram of the shearer fiber optic cable.

To analyze the impact of each feature on the model’s prediction results, this study separates the shearer fiber optic cable into control conductors 1, 2, 3, 4 and power conductors 1, 2, 3, as shown in the cable conductors diagram in Fig 18. This separation method allows for the evaluation of the contribution of each conductor to the model’s prediction results.

Due to the structural and functional relationships between the optical fiber, power conductor, and control conductor in the cable, multicollinearity may arise, which could affect the accuracy and stability of SHAP values. To address this issue, Principal Component Analysis (PCA) was introduced before performing the SHAP analysis to reduce the dimensionality of the input data. PCA minimizes the correlations between features by transforming the original feature space into an orthogonal space, thereby reducing multicollinearity and enhancing the stability and reliability of SHAP value calculations. This processing ensures that the SHAP analysis results are more accurate and effectively assess the impact of each feature on the model’s prediction outcomes.

**Fig 19:**
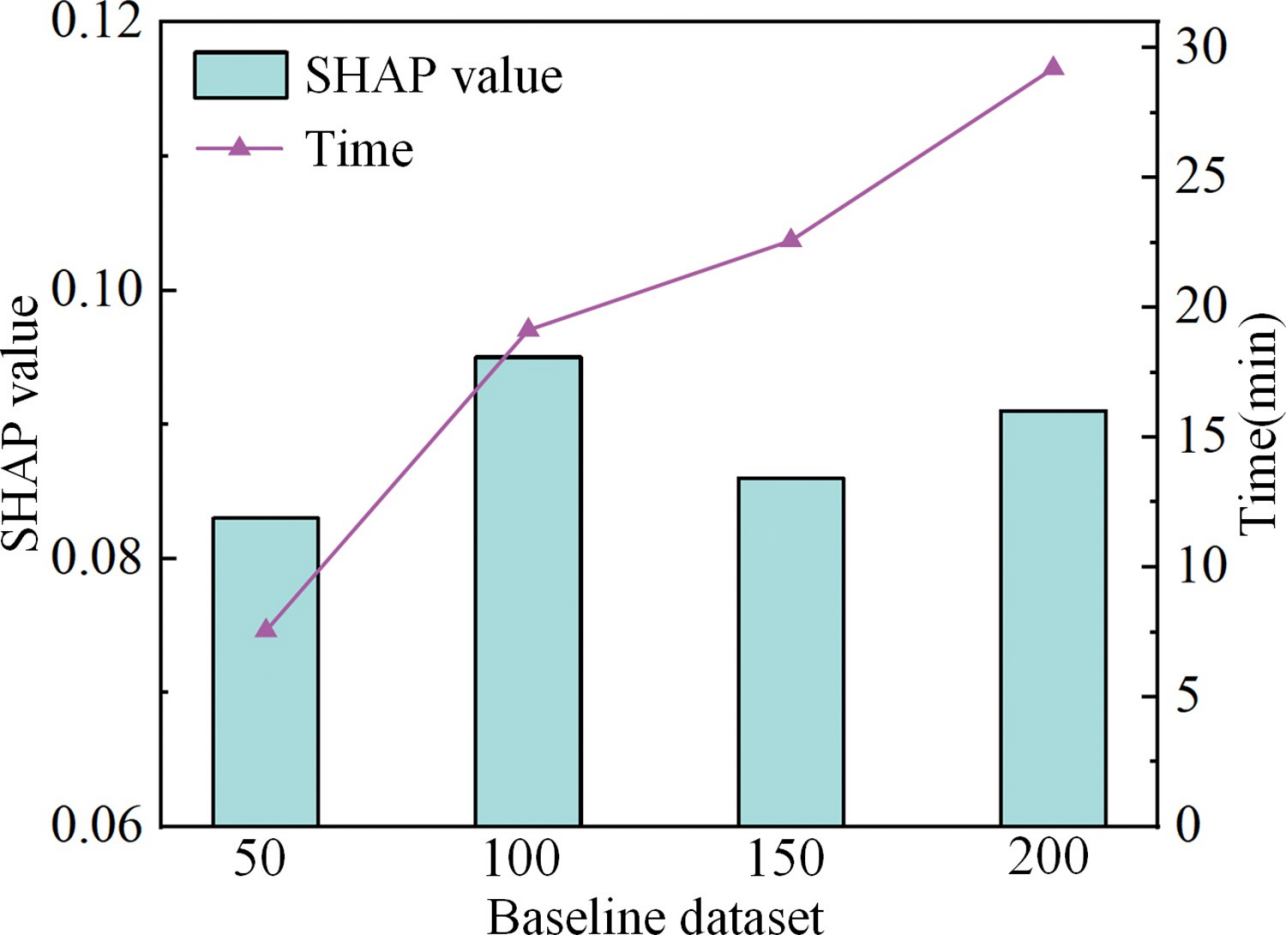
Separation diagram of the shearer fiber optic cable.

To verify the stability of the baseline dataset selection, this study conducted a sensitivity analysis by adjusting the size of the baseline dataset and comparing the effects of using 50, 100, 150, and 200 samples as the baseline dataset on SHAP values, as shown in Fig 19.

As shown in Fig 19, when the background dataset sizes are 50, 100, 150, and 200, the corresponding SHAP values are 0.083, 0.095, 0.086, and 0.091. The results indicate that the size of the baseline dataset has a minimal impact on the SHAP value calculation, further verifying the stability and reliability of the SHAP analysis results. However, as the number of baseline dataset samples increases, the computation time also shows an increasing trend. When the background dataset consists of 200 samples, the computation time reaches 29.22 minutes. Considering both computational efficiency and result stability, 100 samples were ultimately selected as the baseline dataset for subsequent experiments.

**Fig 20:**
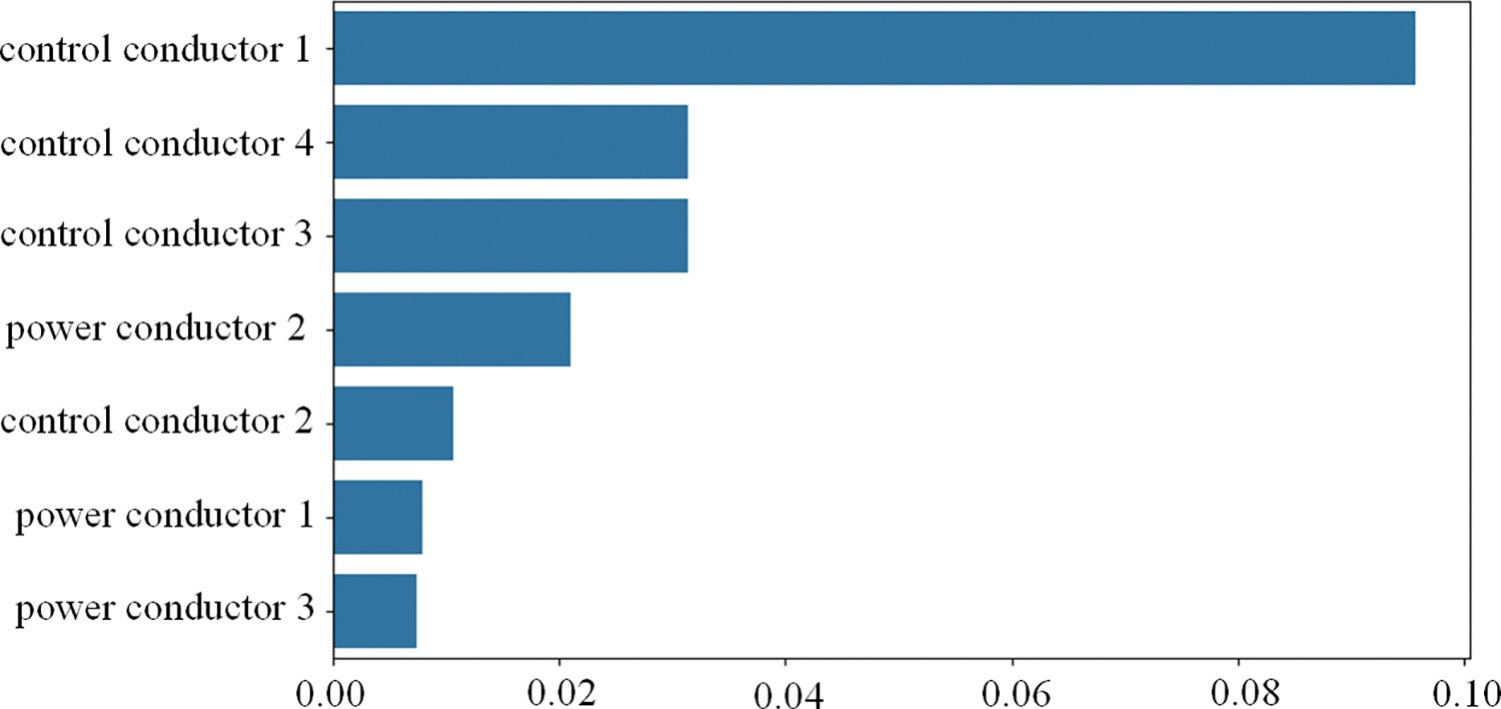
Contribution of individual features.

The contribution of each feature to the fiber optic cable prediction results, based on the SHAP values, is shown in Fig 20. In Fig 20, control conductor 1 has the most significant impact on the model, with a SHAP value of approximately 0.095, indicating that it is the most critical variable in predicting the mechanical properties of the fiber optic cable. In contrast, the cumulative contribution of power conductor 1 and 3 is minimal, with SHAP values of about 0.006 and 0.005, respectively, indicating that they have a relatively small effect on the model’s prediction results. The features of the control conductors are more important than those of the power conductors and play a dominant role in the prediction results.

**Fig 21:**
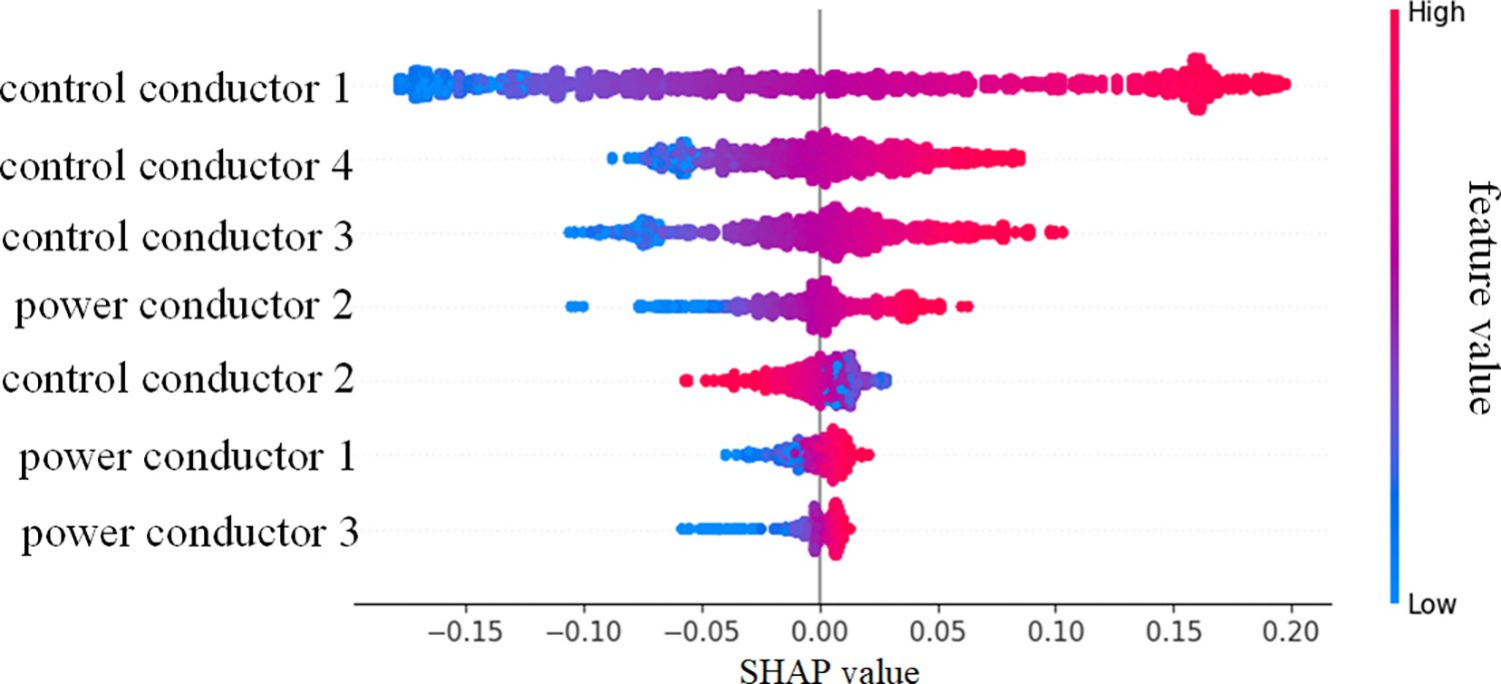
Contribution trends of each feature.

The SHAP feature contribution honeycomb plot illustrates the contribution trends of each feature to the model’s output, as shown in Fig 21. The horizontal axis represents the SHAP value, which indicates the influence of each feature on the model’s output, while the vertical axis lists the features. The color of the scatter points reflects the magnitude of the feature values (red for high values, blue for low values). When the SHAP value is positive, it means the feature positively influences the model’s predicted output, while a negative SHAP value indicates the opposite. In Fig 18, the SHAP value distribution of control conductor 1 has the widest range, and especially at high feature values, it makes a significant positive contribution to the output. This indicates its crucial role in mechanical property prediction, with significant contributions and fluctuations observed both globally and locally, playing a key role in the stability and accuracy of the model’s predictions. Control conductor 3 and 4 have a secondary influence, while the impact of the power conductors is relatively small.

The quantitative analysis of SHAP values highlights the critical role of control conductor features in predicting the mechanical properties of fiber optic cables. As the weakest part of the optical fiber cable, the control conductor is prone to fatigue and failure during operation. Therefore, in the structural design of fiber optic cables, special attention should be given to the control conductors to ensure their stability and minimize their impact on the accuracy of model predictions. This provides a theoretical basis for cable optimization, effectively enhancing the model’s robustness under complex operating conditions and reducing the risk of system failures caused by instability in key variables. Furthermore, by establishing a real-time monitoring system for optical fiber cables and integrating this model for fault prediction, the monitoring efficiency of optical fiber cables can be significantly improved, extending the service life of the equipment and ultimately enhancing the safety and reliability of mining equipment.

## 6. Conclusion

To enhance the reliability and service life of shearer fiber optic cables, this study proposes a hybrid model based on TCN-BiLSTM-SEAttention to achieve high-precision predictions of the mechanical properties of shearer fiber optic cables under bending conditions. Theoretical analysis and experimental validation yield the following conclusions:

The TCN-BiLSTM-SEAttention model combines the temporal feature extraction capability of TCN, the bidirectional information capture ability of BiLSTM, and the adaptive feature optimization capability of SEAttention. The SEAttention mechanism adjusts the feature weights, significantly enhancing the model’s feature representation and prediction accuracy, providing a reliable solution for predicting mechanical properties under complex operating conditions.

The TCN-BiLSTM-SEAttention model performs excellently in all evaluation metrics, with MSE, RMSE, and MAE reduced to 0.0002, 0.0159, and 0.0126, respectively, and R^2^ reaching 0.981. Compared to other predictive models, the prediction accuracy is significantly improved. This provides a more accurate tool for the subsequent assessment and safety management of optical fiber cables, significantly reducing equipment failure rates, improving operational efficiency and safety, and providing strong technical support for the long-term reliability of shearers.

Using the stress data from power conductors, control conductors, and optical fibers as input features, the TCN-BiLSTM-SEAttention model effectively captures the stress relationships between different conductor cores and accurately predicts the stress in the control and power conductors. The maximum prediction errors for the power and control conductors under different bending speeds are 0.56% and 0.86%, respectively.

Based on SHAP feature importance analysis, the control conductor has the highest SHAP value of 0.095, making it the key feature affecting the model’s prediction performance. The prominence of the control conductor feature provides important guidance for future cable monitoring and maintenance, emphasizing the necessity and value of feature selection in practical applications. Future research could integrate composite material optimization designs, particularly improving the material properties and structure of the control conductors to enhance their stress resistance, and combine this with the TCN-BiLSTM-SEAttention model to develop a more efficient monitoring and assessment system.

## List of abbreviations

TCN: Temporal Convolutional Network
LSTM: Long Short-Term Memory
BiLSTM: Bidirectional Long Short-Term Memory
SEAttention: Squeeze-and-Excitation Attention
SHAP: SHapley Additive EXplanations
MSE: Mean Squared Error
RMSE: Root Mean Squared Error
MAE: Mean Absolute Error
R^2^: Coefficient of Determination
RNN: Recurrent Neural Network
GRU: Gated Recurrent Unit
CNN: Convolutional Neural Network
VMD: Variational Mode Decomposition
GRU: Gated Recurrent Unit
SVM: Support Vector Machine
WLS: Weighted Least Squares
YOLO: You Only Look Once
ADSS: All Dielectric Self-Supporting
PCA: Principal Component Analysis
10-fold CV: 10-fold Cross Validation
